# Urban Environment and Outdoor Walking Mobility Among Older Adults With and Without Cognitive Impairment in Singapore: Formative Study

**DOI:** 10.2196/76777

**Published:** 2025-10-27

**Authors:** Yuezhong Liu, Chek Hooi Wong, Noam Shoval, Moon-Ho Ringo Ho, Yin-Leng Theng

**Affiliations:** 1 Ageing Research Institute for Society and Education (ARISE), Nanyang Technological University, #01A - 01 Singapore, Singapore, 637335, Singapore, 65 90679806; 2 Research for Impact, Singapore, Singapore; 3 Department of Geography and the Institute for Urban and Regional Studies, Hebrew University of Jerusalem, Jerusalem, Israel; 4 Wee Kim Wee School of Communication and Information, Nanyang Technological University, Singapore, Singapore

**Keywords:** cognitive impairment, daily life gait speed, gross plot ratio, land use, older adults

## Abstract

**Background:**

There is increasing recognition of the environment’s role in shaping cognitive functioning among older adults.

**Objective:**

This study aimed to examine the relationship between the outdoor mobility of older adults with and without cognitive impairment and the built environment in 3 urban neighborhoods in Singapore, specifically 3 urban, high-density subzones selected a priori.

**Methods:**

Outdoor walking mobility in daily life gait speed (DGS) was collected continuously for 1 week using a previously validated hybrid mobility tracker. Mini-Mental State Examination cut-offs by educational levels were used to differentiate cognitive impairment (CI) and without cognitive impairment (nonCI). The environmental characteristics examined were gross plot ratio and land use. Statistical correlations were used to examine the associations between older adults’ outdoor mobility and built environment for all CI and nonCI groups. Two case examples were also used to provide a location-based heatmap on DGS for 3 consecutive days.

**Results:**

The overall mean DGS was 0.75m/s (SD 0.12) for the nonCI group and 0.73m/s (SD 0.08) for the CI group. The between-group difference (0.02m/s) was below commonly cited thresholds. Exploratory land-use and zone-specific summaries suggested context-dependent variation, such as residential areas (CI: 0.80m/s and nonCI: 0.62m/s). Higher GPR was associated with faster DGS in the nonCI group (*β*=0.04, 95% CI 0.01‐0.07, *P*=.04) and slower DGS in the CI group (*β*=–.13, 95% CI –0.20 to –0.04, *P*=.01). CI participants spent more time in commercial and business zones than nonCI participants, while both groups spent the majority of walking time in residential areas. However, estimates were based on small subsamples and multiple unadjusted comparisons and should be interpreted as hypothesis-generating.

**Conclusions:**

This formative, exploratory study suggests that environmental typologies, particularly GPR and land use, may be associated with differences in mobility patterns between older adults with and without cognitive impairment; subgroup patterns were exploratory and not powered for clinical interpretation. Policy implications include integrating fine-grained environmental metrics into age-friendly urban planning. Clinically, mobility assessments should account for environmental context. Future research should use larger, more diverse, and longitudinal samples to confirm these associations and guide the design of supportive urban environments for cognitively diverse aging populations, noting that findings are context-specific to high-density urban environments. However, given data limitations, unmeasured factors such as comorbidity burden, habitual physical activity, socioeconomic context, and caregiver accompaniment may also contribute to the observed patterns, and findings should be interpreted accordingly.

## Introduction

The interaction between cognitive function, mobility, and the built environment has become an area of increasing research interest, particularly in rapidly urbanizing societies where older adults represent a growing segment of the population. Mobility in daily life is not only a marker of functional independence but is also closely associated with cognitive health outcomes in later life. Previous research has shown that slower gait speed is associated with poorer cognitive performance, increased risk of cognitive decline, and higher dementia risk [1-3]. Conversely, improvements in gait speed have been linked to better cognitive outcomes and reduced risk of decline [4,5].

While daily-life gait speed (DGS) is a well-established and ecologically valid indicator of functional health, mobility can also be characterized using other measures such as step count, walking distance, life-space mobility, and mobility diversity [6-9]. These indicators capture different aspects of how individuals interact with their environments. Among these, DGS is particularly valuable because it reflects the ability to sustain efficient locomotion in real-world contexts, integrating physical capacity, cognitive resources, and environmental demands.

Emerging evidence suggests that environmental factors—such as walkability, street connectivity, and access to destinations—can influence mobility behaviors in older adults [10-12]. Within this context, two environmental typologies are particularly relevant: gross plot ratio (GPR) and land use. GPR is a standard urban planning measure of built density, defined as the ratio of a building’s total floor area to the size of the land parcel on which it sits. Higher GPR values indicate denser, often high-rise environments, while lower values reflect lower-density, low-rise areas. In Singapore, GPR is a central parameter in the Urban Redevelopment Authority’s Master Plan and determines allowable development intensity for residential, commercial, and mixed-use areas [13]. Previous studies have linked urban density to pedestrian movement patterns, environmental complexity, visual enclosure, and sensory load, all of which can influence gait speed and navigation—particularly in older adults with cognitive impairment [14-16].

Land use describes the functional designation of an area, such as residential, commercial, business, or community zones. Land use diversity has been associated with opportunities for social participation, access to services, and variety in walking routes, which can influence both physical activity and cognitive engagement [17-19]. For individuals with cognitive impairment, different land use contexts may impose varying navigational and multitasking demands, affecting mobility performance. For example, busy commercial areas may present greater cognitive load due to crowds and signage, while residential zones may offer more predictable, familiar walking environments.

Despite growing interest in the interplay between cognition, mobility, and the built environment, few studies have examined how specific environmental typologies such as GPR and land use interact with cognitive status to shape real-world walking performance. Existing research often treats mobility outcomes without differentiating by environmental type or applies aggregate environmental measures without considering the potentially divergent effects on individuals with and without cognitive impairment.

This study aims to address this gap by examining how GPR and land use are associated with DGS in older adults with and without cognitive impairment living in Singapore. In this context, DGS is used as an observational indicator of mobility performance in different environmental conditions rather than as a diagnostic screening tool. By linking fine-grained environmental metrics with objective mobility tracking data, this work seeks to generate preliminary insights that can inform the design of age-friendly neighborhoods and guide future research on mobility–environment interactions in cognitively diverse aging populations. As Singapore’s built form is predominantly urban and high-density, this formative analysis focuses on within-city variation (eg, GPR bands and land-use categories) rather than urban-to-rural contrasts. As such, the insights are most transferable to similarly dense city contexts and are not intended to generalize to suburban or rural environments.

## Methods

### Ethical Considerations

This manuscript presents a secondary analysis of a deidentified dataset originally collected under National Healthcare Group Domain Specific Review Board approval (NHG DSRB Ref: 2017/00937) and described in [20]. During the original study, all participants were assessed for capacity to consent prior to enrollment. For individuals without cognitive impairment, written informed consent was obtained following a full explanation of objectives, procedures, potential risks, and benefits. For participants with cognitive impairment, trained staff used plain-language explanations and a teach-back process to assess understanding; when participants were able to consent, written consent was obtained directly. Where capacity was uncertain or absent, consent was obtained from a legally acceptable representative, in accordance with local regulations, with verbal or written assent sought from the participant whenever possible. The current secondary analysis involved no new data collection or contact with participants. Use of the dataset for the present purposes was authorized by the data custodian. No additional ethics review was sought because analyses were conducted exclusively on de-identified data previously collected with ethics approval and explicit consent for research use.

All data used in this analysis were deidentified before access by the study team; direct identifiers were removed and replaced with coded study IDs. Electronic files were stored on secure, access-controlled institutional servers with encryption in transit and at rest, accessible only to authorized personnel. Geospatial outputs are reported as aggregate or smoothed summaries (no exact home locations or personally identifying routes are disclosed). Reporting is at a level designed to prevent reidentification. Data retention and destruction follow institutional and NHG DSRB policies. In the original study [20], participants received modest, IRB-approved compensation to acknowledge their time and transport; no performance-based payments were provided. All data are deidentified and reported in aggregate without individual-level identifiers. Procedures adhered to NHG DSRB research policies, the Belmont Report, and the World Medical Association’s Declaration of Helsinki.

### Study Design

This study is a formative secondary analysis of cross-sectional data from a larger national-level project on urban planning and the design of age-friendly neighborhoods in Singapore. In total, 3 study neighborhoods (each approximately 1 km²) were selected a priori from the western, central, and eastern parts of Singapore based on a high proportion of older adults (aged ≥55 y). The formative designation reflects the preliminary, hypothesis-generating nature of this work, given the relatively small sample size and exploratory focus on identifying relationships between environmental typologies (gross plot ratio and land use) and daily-life gait speed in older adults with and without cognitive impairment. The intention is to provide early insights that can guide the design of future, larger-scale, and potentially longitudinal studies to further investigate causal pathways and generalizability. Given the 1-week observation window and cross-sectional design, temporal ordering cannot be established and causal inference is not warranted; all analyses are interpreted as associative and hypothesis-generating. All three study subzones were urban and high-density by design, reflecting Singapore’s prevailing development pattern. This sampling frame was chosen to maximize environmental variation within dense urban settings (eg, across GPR bands and land-use types) while acknowledging that the absence of suburban or rural areas limits external validity. Accordingly, our inferences are intended as analytical generalizations to comparable high-density cities rather than statistical generalizations to low-density suburban or rural contexts.

### Participants

In total, 33 older community‐dwelling adults, 55 years and above, resided in 3 urban subzones (S1, S2, and S3) in Singapore. For this study, “neighborhood” is defined as a 400 ‐ 1000m buffer around the home. Only those residents who resided in up to a 1 km buffer around the subzone were qualified for participation. Participants put on a wearable hybrid GPS and accelerometer tracker when going outdoors continuously for 7 days [20]. The tracker was designed to capture the wearer’s geospatial data accurately within 15 m (depending on the GPS signal) during outdoor travel or activity to enable the geographic positioning of the participant in longitude and latitude and the travel mode speed. Cognitive status was assessed using the Mini-Mental State Examination (MMSE), administered in person by trained research staff during the original data collection phase of the parent study. Education-adjusted MMSE cut-off scores, based on Singapore-specific normative data [21,22], were used to classify participants into cognitively impaired (CI) and cognitively nonimpaired (nonCI) groups: (1) No formal education: MMSE ≤25; (2) primary education: MMSE ≤27; and (3) secondary education or higher: MMSE ≤29.

Participants scoring above these thresholds were classified as nonCI. All participants were screened for capacity to provide informed consent before enrollment. Those without cognitive impairment provided written consent directly. For participants with cognitive impairment, a simplified explanation of the study was given, and understanding was assessed using a teach-back process. Where independent capacity was lacking, consent was obtained from a legally acceptable representative in accordance with local regulations, and verbal or written assent was sought from the participant whenever possible.

### Dataset

#### Individual Data

Basic sociodemographic, cognitive data, and DGS were collected [12,13]. Standardized indices of comorbidity (eg, count or burden), device-based physical-activity metrics beyond outdoor DGS (eg, step count across all domains), detailed socioeconomic indicators beyond education (eg, income and household assets), and caregiver accompaniment were not collected in the parent study. Daily-life gait speed (DGS, m/s) was the sole objective outdoor mobility indicator. It was computed as the average speed of continuous outdoor walking tracks derived from 1-week hybrid GPS+accelerometer logs, restricting to outdoor ambulation bouts and excluding implausible segments (GPS distance ≤10 km). The estimated average speed was calculated based on a continuous walking track and a GPS distance of less than 10km [23].

#### Gross Plot Ratio and Land Use

**Figure 1.**
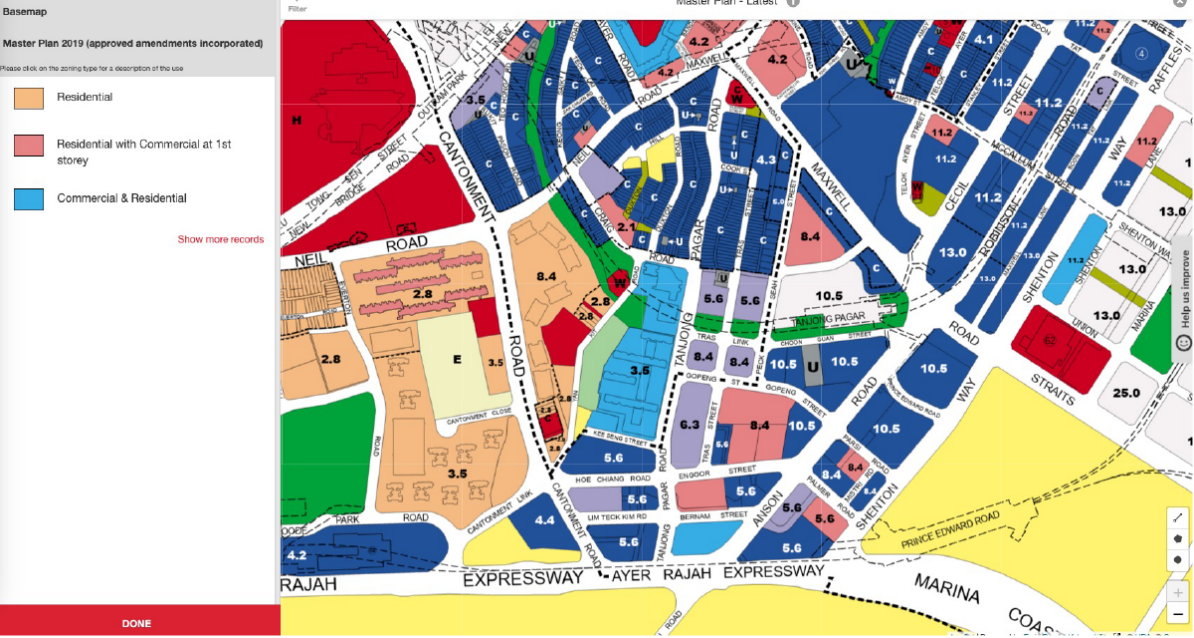
Urban Redevelopment Authority of Singapore (URA’s) online masterplan map with gross plot ratio and land use [27].

The GPR, monitored by the Urban Redevelopment Authority of Singapore (URA), reflects how intensively the land parcel can be used. The GPRs of land parcels are revised in the draft master plan and subsequently, the master plans, along with their respective land uses. Both GPR and land use are used as indicators of development control and land use in different areas of the city in addition to being used for cost forecasting and resourcing a given construction quality [24,25]. URA described the full details of the land use map of Singapore [26]. There are 31 categories of zoning interpretation, such as residential, commercial, hotel, business park, waterbody, and transport facilities. Table 1 shows the general considerations of the development control of GRP and building storeys. If 2 plots of land have similar areas (on the URA Space map Figure 1) but differing plot ratios, it usually means that one will be denser or taller than the other.

**Table 1.** Gross plot ratio categories with corresponding building storeys, as specified in the Urban Redevelopment Authority of Singapore (URA) Master Plan [25].

Gross plot ratio or density	Storeys
1.4 (very low density)	5
1.6 (low density)	12
2.1 (medium density)	24
2.8 (high density)	36
>2.8 (very high density)	>36

### Tracker Platform, Previous Validation, and Postprocessing Pipeline

This paper reports a secondary analysis of data collected with a hybrid GPS–accelerometer tracker developed and validated previously; device specifications, firmware, logging logic, and the original workflow are detailed in [20]. Our present focus is the derivation of DGS from those logs and its association with environmental typologies; readers are referred to [20] for device-level development and validation.

Following the tracker workflow, we implemented a Python pipeline that (1) aligns timestamps between GPS and accelerometer streams by recorded date, (2) applies a short moving-average (3-point) smoothing to latitude or longitude to reduce multipath jitter, (3) computes 1 second ground speed from haversine distances, and (4) removes spikes (ground speed>5.0m/s). Walking versus vehicular mode thresholds followed our a priori rule:<1.39m/s (5km/h)=walking;≥1.39m/s=vehicular.

We defined tracks as sequences of ≥20 consecutive 1 second samples classified as walking (0.30–<1.39m/s), permitting ≤2 second short gaps to bridge brief satellite occlusion, and requiring net displacement ≥20 m to exclude dithering. Any internal spike ≥1.39m/s invalidated the track. For each qualifying track, DGS =total GPS distance / track duration; tracks with GPS distance >10km were excluded as implausible for walking. Accelerometer signals were used to corroborate step-like periodicity within tracks; segments without this periodicity were dropped.

### Data Analyses

Descriptive statistics were used to summarize participant characteristics. Gross plot ratio and land use were retrieved by the public application programming interface from the master plan map [27]. DGS was examined in relation to GPR and land use using Pearson’s correlation and simple linear regression. Before conducting regression analyses, model assumptions of normality, homoscedasticity, and independence were assessed. Normality of residuals was evaluated using Shapiro–Wilk tests and Q–Q plots, while homoscedasticity was examined via residual-versus-fitted plots. Independence of observations was ensured by the study design. For correlations, Pearson *r* values with 95% CIs were reported as measures of effect size. For regression models, standardized *β* coefficients with 95% CIs and corresponding *P* values were presented. Potential confounding variables, including age, sex, and education level, were examined in preliminary analyses, and adjusted models were run where confounding effects were observed. Statistical significance was set at *α*=.05. All analyses were conducted using Python 3.8.0 (*SciPy* 1.9.0 package). Subgroup analyses by land use and GPR were exploratory and not prespecified; *P* values for these comparisons are descriptive and were not adjusted for multiplicity. We therefore emphasize effect sizes and 95% CIs and interpret subgroup findings cautiously. Given the modest sample size, we limited covariate adjustment to a parsimonious set (age, sex, and education) to avoid model overfitting. Additional covariates (eg, comorbidities, broader physical-activity metrics, detailed socioeconomic status, SES, caregiver accompaniment) were unavailable. As a coarse SES descriptor, housing type was highly skewed (public housing in 95% of participants), precluding stable adjustment and limiting SES variability. Accordingly, residual confounding by unmeasured factors is likely, and effect estimates should be interpreted as associative.

## Results

### Participants’ Characteristics

Of 33 participants, 18 were from S1, 8 were from S2, and 7 were from S3. Sociodemographics are shown in Table 2. The mean (SD) age of the samples was 69.2 (7.14) years, 21 of them (21/33, 64%) were females, and the majority were Chinese (31/33, 94%). Half were married, with only 42% (14/33) having completed secondary school or higher in education. The average number of schooling was 7.3 (4.19) years. None of the participants had full‐time employment, with 18% (6/33) holding part‐time employment. The majority of participants lived in public housing (31/33, 95%) and lived in their respective subzones for an average of 23.1 (SD 15.2) years. All participants had no previous clinical diagnosis of dementia.

**Table 2.** Includes age, sex, ethnicity, education, housing type, years of schooling, retirement duration, length of residence, MMSE^a^ score, and cognitive status classification.

Demographics	S1 (n=18)	S2 (n=8)	S3 (n=7)
Age, mean (SD)	71.4 (6.43)	63.3 (4.86)	70.0 (8.00)
Female, n (%)	14 (77.9)	4 (50)	3 (42.9)
Ethnicity, n (%)			
Chinese	18 (100)	6 (75)	7 (100)
Malay	0	2 (25)	0
Highest education completed, n (%)			
No formal education	1 (5.6)	2 (25.0)	0
Primary school	2 (50.0)	3 (37.5)	3 (42.9)
Secondary school	4 (22.2)	2 (25.0)	2 (28.6)
Postsecondary	1 (5.6)	1 (12.5)	1 (14.3)
Tertiary	3 (16.7)	0	0
Do not know or not sure	0	0	1 (14.3)
Housing type, n (%)			
HDB^b^ 1‐2 room	3 (16.7)	0	0
HDB 3 room	10 (55.6)	0	5 (71.4)
HDB 4 room	3 (16.7)	6 (75.0)	0
HDB 5 room or HUDC or executive flat	1 (5.6)	2 (25.0)	2 (28.6)
Condo or apartment^c^	1 (5.6)	0	0
Number of years of schooling, median (SD)	6 (0‐16)	7 (0‐13)	6 (3‐10)
Number of years retired, median (range)	16 (8‐30)	2 (1‐9)	6 (1‐22)
Number of years living here, median (range)	20 (0.5‐50)	20 (8‐32)	25 (4‐50)
MMSE score, mean (SD) (max 30)	27.2 (2.32)	26.4 (2.13)	26.6 (2.22)
Cognitive impairment (MMSE^a^ cut‐offs), n (%)	6 (33.3)	4 (50.0)	4 (57.1)

a MMSE: Mini-Mental State Examination.

b HDB: Housing and Development Board is Singapore’s Public Housing.

c Condo/Apartment is Private housing.

### Outdoor Mobility by Cognitive Impaired and Nonimpaired Participants

The travel mode of the participant was determined by travel speed. A travel speed less than 1.39 m/s (5 km/h) was categorized as walking mode, and a speed above 1.39 m/s (5 km/h) was categorized as vehicular mode [28,29]. Neighborhoods were defined as the respective subzones and a 1 km buffer [30]. Table 2 shows all GPS records (a total of 5,583,089 records per second). Overall, the DGS is 0.74 m/s (2.66 km/h). Across all participants, mean DGS was 0.74 m/s (SD 0.11); by cognitive status, nonCI: 0.75 m/s (SD 0.12) and CI: 0.73 m/s (SD 0.08).

### Outdoor Mobility With Gross Plot Ratio and Land Use

As shown in Table 3, based on one-way ANOVA tests, both groups’ DGS was significantly associated with GPR: nonCI group (*F*=77.13, *P*<.001) and CI group (*F*=111.39, *P*<.001). In correlation analyses, higher GPR was moderately positively correlated with faster outdoor DGS in the nonCI group (*r*=0.69, 95% CI 0.28-0.88, *P*=.003) and strongly negatively correlated with DGS in the CI group (*r*=–0.75, 95% CI –0.91 to ‐0.38, *P*=.001).

**Table 3.** Association between gross plot ratio (GPR) and daily-life gait speed (DGS) for cognitively nonimpaired (nonCI) and cognitively impaired (CI) participants.

Group	Mean DGS^a^ (m/s)	SD DGS (m/s)	GPR-DGS,^b^ *β*	GPR-DGS, R	95% CI	*P* value	*F* statistic (ANOVA)	Model *R*²
NonCI^c^	0.75	0.12	0.04	0.69	0.01 to 0.07	.03	77.13	0.48
CI^c^	0.73	0.08	–0.13	–0.75	–0.20 to –0.05	.01	111.39	0.56

a DGS: daily life gait speed.

b GPR: gross plot ratio.

c CI: cognitive impairment.

Within this urban frame, exposure variability was present across both land-use categories (residential, commercial, business, and community) and GPR levels ranging from 2.1 to 5.6 (Table 4), enabling within-city contrasts despite the absence of suburban or rural environments. In unadjusted linear regression models, a one-unit increase in GPR was associated with a 0.04 m/s faster DGS in the nonCI group (standardized *β*=.42, 95% CI 0.01-0.07, *P*=.03, R²=0.47, adjusted *R*²=0.44) and a 0.13m/s slower DGS in the CI group (standardized *β*=–.55, 95% CI –0.21 to ‐0.05, *P*=.01, *R*²=0.56, adjusted *R*²=0.53). After adjusting for age, sex, and education level, the associations remained statistically significant for both groups (nonCI: *β*=.40, 95% CI 0.01-0.07, *P*=.04, *R*²=0.49, adjusted *R*²=0.45; CI: *β*=–.53, 95% CI –0.20 to ‐0.04, *P*=.01, *R*²=0.58, adjusted *R*²=0.54).

**Table 4.** Daily-life gait speed (DGS) and time spent across gross plot ratio (GPR) categories and land use types for cognitively nonimpaired (nonCI) and cognitively impaired (CI) participants.

Category (GPR and land use types)	Mean DGS (m/s), NonCI	Mean DGS (m/s), CI	Time spent (min/wk), NonCI	Time spent (min/wk), CI
GPR				
2.10	0.69	0.81	0.95	0.00
2.50	0.71	0.78	3.55	0.00
2.80	0.73	0.76	106.20	49.25
3.00	0.75	0.74	163.32	15.57
3.20	0.77	0.72	0.58	0.00
3.50	0.78	0.70	5.37	0.35
4.00	0.80	0.68	11.65	3.33
4.20	0.81	0.66	19.42	4.60
4.50	0.82	0.65	0.72	0.30
4.90	0.83	0.64	0.30	0.00
5.60	0.84	0.63	0.23	0.00
Land use				
Community	0.62	0.8	2214.70	1531.00
Commercial	0.76	0.67	2.20	4.60
Business	0.72	0.65	8.05	24.08
Residential	0.67	0.70	54.05	258.30

When stratified by land use typology, effect sizes varied. In business zones, the nonCI group walked faster than the CI group (mean difference=0.07 m/s, 95% CI 0.03-0.11, *P*=.002, Cohen *d*=0.84). In commercial zones, a similar pattern was observed (mean difference=0.09 m/s, 95% CI 0.04-0.13, *P*<.001, Cohen *d*=0.92). Conversely, in community and residential zones, CI participants walked faster than nonCI participants (community: mean difference=–0.03 m/s, 95% CI –0.05 to ‐0.01, *P*=.02, Cohen *d*=–0.58; residential: mean difference=–0.18 m/s, 95% CI –0.23 to ‐0.12, *P*<.001, Cohen *d*=–1.21). When stratified by land-use typology, numerical differences varied across contexts. Several comparisons reached nominal statistical significance (eg, business and commercial zones), but these subgroup analyses were exploratory, based on small cell sizes, and not adjusted for multiple testing. CI indicated limited precision in several strata. Accordingly, these results are hypothesis-generating and should not be interpreted as clinically meaningful without replication.

These findings indicate that both GPR and land use are associated with variations in DGS, and that the direction and magnitude of these associations differ by cognitive status.

Table 4 presents the distribution of mean DGS and time spent across each GPR category and land use type. In addition to descriptive summaries, group comparisons were conducted to examine differences in time spent across each GPR category and land use type. Due to nonnormal distributions, Mann–Whitney *U* tests were applied. No significant differences were found in time spent across GPR categories between CI and nonCI participants (all *P*>.05). For land use types, CI participants spent significantly more time in commercial areas (median [IQR]: 198 [140–256] min/week) compared to nonCI participants (154 [112–196] min/wk, *P*=.04), and more time in business areas (112 [84–145] vs 86 [65–108] min/wk, *P*=.03). Both groups spent the majority of their walking time in residential areas, with nonCI participants spending more time than CI participants (2214.7 vs 1531.0min/wk, *P*=.02). Time spent walking in community zones was minimal for both groups (<5% of total walking time).

## Discussion

### Daily Life Gait Speed (DGS) With Built Environment

There are significant differences in the mobility of CI and nonCI samples between DGS and built environment variables (gross plot ratio and land use) in Tables3  4. Cognition is associated with variations in outdoor mobility among older adults in the community. Cognition, including executive functions, has been associated with the ability to perform concurrent motor and cognitive tasks during outdoor navigation in older adults [31,32]. Lamoth et al observed that differences in cognitive function co-occur with differences in gait variability, stability, and dual-task walking [32]. There were small differences in DGS for both nonCI (0.75 m/s) and CI (0.73 m/s). While the overall mean difference in DGS between CI (0.73 m/s) and nonCI (0.75 m/s) participants was 0.02m/s, this value falls below the commonly cited minimal clinically important difference (MCID) for gait speed in older adults, which ranges from approximately 0.05 m/s for a small meaningful change to 0.10 m/s for a substantial change [33,34]. Therefore, this overall difference should not be interpreted as clinically significant in isolation, exploratory stratification by environmental typology yielded larger numerical differences in some contexts (eg,0.18 m/s in residential zones). However, MCID estimates were derived primarily from clinic-based tests and may not translate directly to ecologically measured daily-life gait speed; moreover, subgroup sample sizes were small, and comparisons were not adjusted for multiplicity. Therefore, these patterns should be viewed as preliminary signals requiring confirmation rather than as evidence of clinically meaningful subgroup effects. This pattern is consistent with context-dependent differences in gait speed between older adults with and without cognitive impairment, underscoring the value of examining mobility within specific environmental settings rather than relying solely on aggregate averages. When stratified by land use, we observed larger differences in DGS. On average, nonCI older adults were observed to walk more slowly than CI older adults in low-GPR (lower-density) areas and in community and residential zones, whereas nonCI older adults were observed to walk more quickly in high-GPR (higher-density) areas and in business and commercial zones. The findings were consistent for both GPR and land use results, as lower GPR is approved for community and residential use, and high GPR is approved for business and commercial use.

Alternative explanations and residual confounding. While models adjusted for age, sex, and education, other factors not measured in this study - such as comorbidity burden, habitual physical activity beyond outdoor DGS, socioeconomic conditions beyond education, and caregiver accompaniment - could be associated with both where and how participants walk and with observed gait speed. Given the modest sample and lack of harmonized measures for these domains, we could not control for them directly; thus, the observed associations with GPR and land use should be interpreted with caution as they may partly reflect unmeasured confounding.

Both groups spent the majority of their time walking in residential areas (nonCI: 2214.7 min, CI: 1531.0 min), with nonCI spending more time walking. We postulate that CI and nonCI individuals may have different walking behaviors while interacting with different land uses. Various studies have shown that standardized measures such as lower extremity strength and indoor gait speed [35] are important for older adults to interact with their physical environments. However, the measures represent a physiological potential in mobilizing in the community. Outdoor walking speed is more reflective of real-world interactions with the environment, which may be influenced by cultural, economic, and social factors [36-38]. While our data indicate that CI participants walked faster in community zones than nonCI participants, this finding should be interpreted with caution, as community walking comprised a very small proportion of total walking time for both groups. The limited exposure to this land use type may not provide a robust basis for generalizing speed patterns in community environments. The additional analyses also revealed that CI participants spent more time in commercial and business zones than nonCI participants, a pattern that co-occurred with the gait-speed differences observed in these contexts. As these observations were post hoc, based on small strata, and not adjusted for multiple comparisons, they are exploratory and hypothesis-generating only.

The lower DGS observed in nonCI participants within community and residential land use areas may be associated with higher levels of interaction and participation with activities in those environments. In Singapore, residential zones often include integrated amenities such as shops, markets, and community centers, which could influence walking speed by encouraging frequent stops or interactions. However, we did not measure cultural, economic, or social participation in this study, and any such explanations remain speculative. Future research that incorporates these contextual variables is needed to assess their potential influence on mobility patterns.

Older adults with CI walked with a lower DGS in business and commercial areas. One unmeasured, hypothesis-generating explanation is greater cognitive or multitasking demands in these environments; however, these factors were not quantified in this study. Furthermore, these potential explanations were not directly measured and should be considered hypotheses for future study rather than confirmed effects. Studies have also reported both positive and negative effects of the gross plot ratio on DGS [36-38]. For example, higher-density built forms may provide greater shade that could support thermal comfort [39]. Prominent buildings could assist wayfinding and orientation [40]. Conversely, tall-building forms may create wind conditions and visual enclosure that could impede comfort or perceived effort [41]. We did not measure microclimate, wayfinding, or wind conditions; these potential mechanisms remain hypotheses for future study. The higher DGS among nonCI participants in business and commercial areas may be related to the environmental features noted above, such as improved wayfinding or shading in high-density zones. As these environmental influences were not directly quantified in our dataset, they remain speculative and warrant further investigation.

### External Validity and Context

Our findings should be interpreted in light of the setting: three a priori selected, urban, high-density subzones in Singapore. While this design provided fine-grained contrasts across land use and GPR bands within dense neighborhoods, it does not capture suburban or rural exposures. Consequently, the results are context-specific and best transferred to similarly high-density city environments. Extending external validity will require multisite designs that deliberately sample lower-density neighborhoods (eg, suburban towns) and rural settings, and that incorporate cross-city comparisons where urban morphology, transport supply, and climatic conditions differ.

### Case Example of DGS Between CI and NonCI Individuals in the Same Neighborhood

To illustrate potential differences in mobility patterns between older adults with and without cognitive impairment, we examined one participant from each group who lived in the same subzone, were of similar age (CI: 82 y; nonCI: 77 y), and had comparable familiarity with the neighborhood. These participants were selected for illustrative purposes only, and the findings are not intended to be representative of their respective groups.

The CI participant (male, MMSE score: 23) walked a total of 8.4km over three consecutive monitored days, with an average DGS of 0.76 m/s. The majority of walking time (72%) occurred in residential areas, followed by commercial zones (18%) and community zones (10%). The nonCI participant (female, MMSE score: 26) walked 10.2 km over the same period, with an average DGS of 0.82 m/s, and a more varied distribution of walking time: residential areas (58%), commercial zones (28%), and community zones (14%).

**Figure 2.**
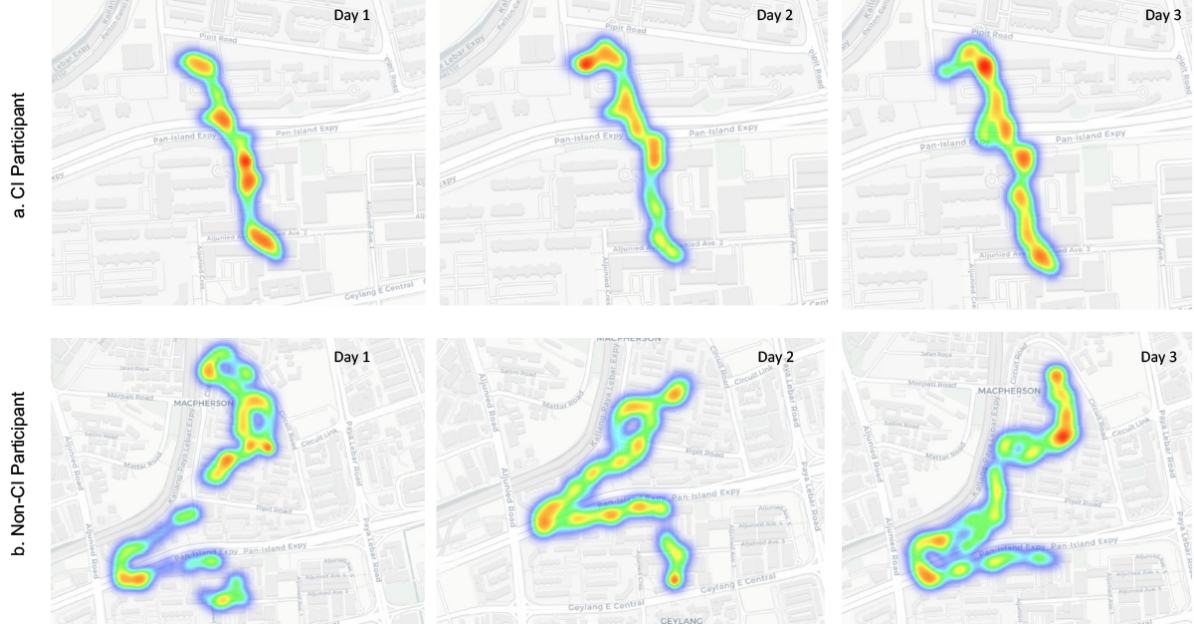
Daily outdoor trajectory comparison of two cases (CI and nonCI) living in the same area (color represents the daily life gait speed; blue represents low speed and red represents high speed, average DGS is 0.74m/s). CI: cognitive impairment.

Heatmaps (Figure 2) show that the CI participant’s walking routes were predominantly linear and repetitive, concentrated along a familiar daily route, with relatively consistent gait speeds (more red areas) along the same path. In contrast, the nonCI participant’s routes were more varied and nonlinear, covering a wider range of neighborhood locations with fluctuating walking speeds.

Data on whether participants walked alone or with a caregiver were not collected; therefore, potential influences of accompaniment on route choice, walking speed, and environmental engagement could not be assessed. This represents an important area for future research. The case study findings should be interpreted as exploratory and hypothesis-generating, providing a visual and quantitative example of how mobility patterns may differ between CI and nonCI individuals when navigating the same neighborhood environment.

Studies have also shown that familiarity with the environment may be associated with differences in DGS and the extent of travel [42,43], especially for persons with dementia [44]. These patterns suggest that mobility differences between CI and nonCI groups are context-dependent and may be influenced by environmental demands as well as individual capabilities. However, given the preliminary and exploratory nature of this work, these observations should be interpreted with caution. Individuals who are familiar with their surroundings tend to navigate more confidently and efficiently, which can influence their walking speed. While these case examples illustrate potential influences of environmental familiarity on gait speed, they are descriptive and based on a small number of participants. As such, they serve to generate hypotheses rather than confirm causal relationships.

Taken together, our findings provide early evidence that built environment typologies and cognitive status may be linked to differences in outdoor walking patterns. Nonetheless, several methodological and contextual limitations should be considered when interpreting these results.

### Limitations and Conclusions

Within these constraints, this formative study provides preliminary, within-city evidence that environmental typologies (GPR and land use) are associated with differences in daily-life gait speed among older adults in high-density urban neighborhoods. This study has several limitations that should be considered when interpreting the findings. First, the sample size was modest (n=33), with participants further divided into CI and nonCI groups, which reduced statistical power and may have limited our ability to detect subtle subgroup differences, particularly when stratified by land use or gross plot ratio categories. Second, recruitment was limited to three urban, high-density subzones in Singapore that were selected for having a high proportion of older adults, which may introduce selection bias and constrain the range of environmental exposures. Third, the geographic context is specific to Singapore’s high-density urban environment; findings may not be directly generalizable to lower-density suburban or rural regions with different built forms, transport systems, and cultural or climatic conditions. Fourth, the study’s cross-sectional design restricts the ability to establish temporal or causal relationships between cognitive status, mobility patterns, and environmental typologies. Observed associations should therefore be interpreted as indicative rather than confirmatory. Fifth, we lacked harmonized measures of key potential confounders—including comorbidities, broader physical-activity metrics (eg, device-based step counts across domains), socioeconomic indicators beyond education, and caregiver accompaniment—which may influence both environmental exposure and mobility. Because housing type was heavily skewed toward public housing (95%), SES variation was limited and not amenable to stable adjustment. Consequently, residual confounding remains a plausible explanation for part of the observed associations. In addition, subgroup analyses (by land use and GPR) were exploratory, underpowered, and not multiplicity-adjusted; consequently, nominal *P* values are descriptive and clinical interpretation of subgroup contrasts is not warranted.

Within these constraints, this formative, exploratory study examined how built-environment typologies were associated with daily-life gait speed. The overall group difference was small and below commonly cited MCID thresholds. Although exploratory stratification revealed larger numerical differences in some settings (eg, 0.18 m/s in residential zones), these subgroup contrasts were based on small cell sizes and were not adjusted for multiple testing; their clinical relevance is uncertain and requires replication. These findings are consistent with context-dependent differences in mobility patterns between CI and nonCI groups; given the small sample and cross-sectional, 1-week design, they should be interpreted as preliminary and associational. In light of unmeasured comorbidity, physical activity, socioeconomic, and caregiving factors, these findings should be viewed as preliminary and associative rather than explanatory of underlying mechanisms. Further research with larger, more diverse cohorts and longitudinal tracking is needed to confirm these patterns, explore potential mechanisms, and inform the design of urban environments that support mobility for older adults across cognitive profiles.

From a policy perspective, our results highlight the value of incorporating fine-grained environmental measures, such as gross plot ratio and land use diversity, into urban design guidelines to support mobility and accessibility for older adults, particularly those with cognitive impairment. For clinical practice, we recommend that gait speed assessments and mobility interventions consider the specific environmental contexts in which walking occurs, as some settings (eg, high-density commercial areas) may impose greater cognitive or navigational demands. To enhance external validity, future research should adopt multisite, stratified sampling across density gradients (including suburban and rural settings) and across cities with distinct urban forms, thereby testing the transferability of these associations beyond high-density contexts. By aligning research, practice, and policy efforts, urban environments can be better designed to promote safe, accessible, and supportive mobility for older adults across cognitive profiles.

## Abbreviations

CI: cognitive impairment
DGS: daily life gait speed
GPR: gross plot ratio
MMSE: Mini-Mental State Examination
NHG-DSRB: National Healthcare Group Domain Specific Review Board
URA: Urban Redevelopment Authority
